# Special Issue “Advances in Molecular Research on Autoimmune Diseases”

**DOI:** 10.3390/ijms252111487

**Published:** 2024-10-25

**Authors:** Davide Cossu

**Affiliations:** 1 Department of Neurology, Juntendo University, Tokyo 1138431, Japan; davide@juntendo.ac.jp; 2 Biomedical Research Core Facilities, Juntendo University, Tokyo 1138431, Japan; 3 Department of Biomedical Sciences, Sassari University, 07100 Sassari, Italy

Autoimmune diseases represent a diverse array of disorders in which the immune system mistakenly attacks the body’s own cells and tissues [1]. These conditions often target various organs, leading to significant disability, increased morbidity, and diminished quality of life [2]. Despite the multifaceted nature of autoimmune diseases, recent advancements have shed light on their underlying mechanisms, with a growing focus on genetic predispositions [3], environmental triggers [4], and immune dysregulation [5].

Recent research highlights the central role that genetic factors play in modulating immune responses [6]. Numerous susceptibility genes have been identified, revealing how they interact with environmental factors, such as infections, to either trigger or exacerbate autoimmune conditions [7]. However, genetic predisposition alone is rarely sufficient to initiate disease; instead, the disruption of immune homeostasis and the persistence of inflammatory responses are the key drivers of autoimmune pathogenesis [8].

Technological advancements, particularly in the use of animal models [9] and high-throughput screening techniques [10], have greatly expanded our understanding of immune pathways involved in autoimmune diseases.

For instance, the experimental autoimmune encephalomyelitis (EAE) model has been instrumental in studying neuroinflammatory conditions such as multiple sclerosis (MS) [11]. These models have revealed specific immune cells, particularly T cells and B cells, as central players in the immune attack against self-antigens [12]. Furthermore, the identification of autoantibodies targeting specific proteins has provided valuable biomarkers for both the diagnosis and prognosis of autoimmune diseases like MS [13] and systemic lupus erythematosus (SLE) [14].

This Special Issue builds upon recent research progress by focusing on the immunological mechanisms underlying a wide range of autoimmune disorders. The issue presents a collection of original research articles examining conditions such as autoimmune thyroiditis, rheumatoid arthritis [15], SLE [16], primary biliary cholangitis [17], and mixed connective tissue disease [18].

Notably, several studies focus on autoimmune diseases affecting the central nervous system (CNS). Two experimental studies leverage the EAE model of neuroinflammation, while a retrospective clinical study offers valuable insights into human disease patterns.

One of the featured studies, led by Zivković et al., explored the role of the growth hormone (GH) axis during the acute disease phase of monophasic EAE in female Dark Agouti rats [19]. While the expression of growth hormone-releasing hormone and somatostatin remained stable in the hypothalamus, significant changes were detected in other regions. In the pituitary gland, genes encoding GH, its receptor, and GH-releasing hormone were significantly upregulated. Despite this genetic shift, the volume of somatotropic cells in the pituitary remained unchanged, though serum GH levels increased and insulin-like growth factor 1 (IGF-1) concentrations dropped, suggesting a state similar to GH resistance. This condition is believed to result from inadequate nutrient intake during the peak of CNS inflammation. The findings suggest that the GH/IGF-1 axis could serve as a therapeutic target in both EAE and MS, with the potential to influence disease progression.

Another study explored the effects of endurance exercise on EAE induced by the myelin oligodendrocyte glycoprotein peptide in NOD/ShiLtJ mice, modeling secondary progressive MS [20]. The researchers found that initiating exercise after disease onset significantly attenuated disease severity, coinciding with a reduction in B cells, dendritic cells, and neutrophils in the CNS. Additionally, increased expression of major histocompatibility complex class II and alterations in costimulatory molecules on CNS B cells and dendritic cells were observed. However, no significant changes in T cell responses were detected in either the CNS or peripheral tissues. These findings suggest that exercise may modulate disease progression by influencing the innate immune system, offering a promising therapeutic strategy for established secondary progressive EAE.

A separate study examined the viral etiology of CNS autoimmune diseases, focusing on the role of viral infections in MS, neuromyelitis optica spectrum disorder, and myelin oligodendrocyte glycoprotein-antibody disease [21]. In a retrospective analysis involving Japanese patients and healthy controls, researchers used a peptide-based enzyme-linked immunosorbent assay (ELISA) to detect specific antibody responses. The study found a marked increase in antibody responses against a peptide derived from the Epstein–Barr virus (EBV) nuclear antigen 1 (EBNA1) in MS patients [22], aligning with prior studies that suggest a strong association between EBV infection and the onset of MS [23]. Interestingly, a subset of antibody-positive MS patients showed a correlation between antibodies against EBV peptides and peptides from the human endogenous retrovirus-W (HERV-W) family [24]. This association suggests a mechanism by which EBV reactivation may trigger HERV-W elements, contributing to MS pathogenesis by amplifying autoimmune responses [25]. These findings emphasize the potential role of viral triggers, such as EBV, in initiating or exacerbating autoimmune diseases through the activation of endogenous retroviral elements.

In another study focusing on rheumatoid arthritis (RA), researchers explored the connection between the human microbiome and RA pathology [26]. The study focused on microbial enzymes, particularly peptidylarginine deiminases (PADs), which can citrullinate proteins, leading to the formation of anti-citrullinated protein antibodies [27]. The researchers examined 17 microbial strains to identify homologs of PADs or arginine deiminases (ADs) and tested these for reactivity to anti-citrullinated protein antibodies from RA patients. Using Western blot, ELISA, and statistical analysis, they found that members of the Firmicutes and Proteobacteria phyla carried PAD/AD homologs and citrullinated antigens, which could potentially trigger immune responses in RA patients. These findings offer a molecular link between microbiome dysbiosis and RA pathogenesis.

Another study delved into genetic factors that may influence inflammation and iron metabolism in RA and SLE [28]. Researchers examined the frequency of the IL-6 promoter polymorphism (−174G>C) and mutations in the HFE gene, which are associated with iron overload [29], in a cohort of Italian patients. The findings pointed to a decreased frequency of both the IL-6 polymorphism and the p.Cys282Tyr mutation in RA and SLE patients, suggesting a potential role in disease susceptibility.

Both studies highlight the complex interplay between host genetics, microbial factors, and immune dysregulation in autoimmune diseases. The identification of IL-6 polymorphisms and microbial PAD homologs offers insights into complementary pathways that may drive autoimmunity in diseases like RA.

Further, autoimmune thyroiditis (AIT), the leading cause of primary hypothyroidism and one of the most common organ-specific autoimmune conditions [30], was explored through a genetic analysis conducted on 237 AIT patients of Polish origin [31]. Contrary to findings in Japanese populations [32], the study found no significant differences in the genotype or allele frequencies of TPO gene promoter polymorphisms compared to healthy controls, highlighting the potential role of ethnic and genetic diversity in the susceptibility to autoimmune diseases like AIT.

Lastly, two important autoimmune diseases—primary biliary cholangitis (PBC) and mixed connective tissue disease (MCTD)—were explored. A study on PBC, a chronic autoimmune liver disease leading to intrahepatic bile duct destruction [17], assessed the diagnostic value of serum Circulating Monocyte Chemoattractant Protein-1 (MCP-1), showing elevated levels in 66% of PBC patients and suggesting that MCP-1 may serve as a biomarker for liver fibrosis progression [33]. Another study analyzed global DNA methylation differences in MCTD [34], SLE, and systemic sclerosis patients, revealing distinct methylation profiles that highlight the role of epigenetics in autoimmune pathogenesis [35].

Both studies add significant knowledge to our understanding of the immune mechanisms underlying autoimmune diseases. While MCP-1 emerges as a promising marker for liver fibrosis in PBC, the role of global DNA methylation in MCTD offers a fresh perspective on the epigenetic contributions to disease progression. Future research integrating these biomarkers with clinical parameters may lead to improved diagnostic tools and therapeutic approaches for these challenging diseases.

Collectively, this Special Issue presents a wealth of scientific insights, advancing our understanding of the mechanisms that drive autoimmune diseases and highlighting potential therapeutic targets. As the intricate interplay between the immune and nervous systems continues to unfold, cross-disciplinary approaches will be essential in developing more effective treatments for these complex and debilitating diseases.
